# Expert Versus Novice: Criminal Expertise in Sexual Burglary and Sexual Robbery

**DOI:** 10.1177/10790632211024236

**Published:** 2021-06-18

**Authors:** Kylie S. Reale, Eric Beauregard, Julien Chopin

**Affiliations:** 1 Simon Fraser University, Burnaby, British Columbia, Canada

**Keywords:** offense behavior, crime-commission process, offense skills, sexual offending

## Abstract

Although there has been considerable variation in the application of expertise to offending populations, one aspect that is widely agreed upon is that expertise is best represented on a continuum from novice to expert. The present study, therefore, investigated criminal expertise in 877 hybrid offenses that involve sexual assault and robbery (i.e., sexual robbery) or burglary (i.e., sexual burglary). Specifically, we analyzed the crime-commission processes of both these offenses using latent class analyses to determine the heterogeneity of criminal expertise among each domain. Results showed an expert to novice continuum in both domains, including a “domain-specific” expert sexual burglary subgroup who was characterized by a high degree of offense-related competencies relevant to sexual burglary. We also found an expert subgroup in sexual robbery who had more general skills (i.e., overlapping expertise) relevant to violent offending. Implications for offender decision-making, treatment, and practice are discussed.

The label of “expert” is typically reserved for a person who has superior skills within a specific domain and the ability to consistently perform at an exceptionally high level (Bourke et al., 2012). Although most research to date has examined “experts” in socially acceptable domains, such as sports or academia, in more recent years, researchers have become interested in the application of functional expertise (i.e., “criminal expertise”) to offender groups (see Nee & Ward, 2015, for a review). Functional expertise is much appropriate for criminal domains because it can be developed over much shorter periods of time, or through indirect means such as covert and symbolic modeling and mental rehearsal (Ó Ciardha, 2015; Ward, 1999). Thus, “criminal expertise” does not necessarily mean that a person has an extensive or “specialized” criminal career, but rather, captures an expert offender’s offense related competency relative to that of a novice (e.g., Bourke et al., 2012; Nee & Ward, 2015; Reale et al., in press; Ward, 1999). In support of this perspective, studies have found that some offenders develop expertise in their specific offense domain, enabling them to become quicker and better at acting on offense-related cues than more novice offenders (Nee, 2015). As such, Nee and Ward (2015) proposed that a more appropriate label for functional expertise within criminal domains is “dysfunctional expertise,” given the potential outcome (i.e., successfully committing a crime).

Although several advancements to the expertise literature have been made through an examination of burglary (see Nee, 2015 for a review), and more recently in sexual offending (Bourke et al., 2012; Chopin et al., 2021), more complex crimes such as those that combine two specific types of offenses (i.e., a hybrid offense) have been largely overlooked in both the criminal expertise literature and in criminological studies more generally (Beauregard & Chopin, 2020). In the first study of criminal expertise on hybrid offenders, Reale et al. (in press) showed that the crime-commission process of sexual burglary (i.e., break-and entering, theft, and sexual assault offense) involved more “domain-specific” expertise in detection avoidance, compared with sexual robbery (i.e., personal theft and sexual assault offense), but also found similar skills related to target appraisal between these two offenses, suggesting these offenses may also share an “overlapping expertise” or “transferable” expertise (Nee et al., 2019) related to violent offending.

This study, however, did not examine the heterogeneity of expertise within each of these domains. This is important because understanding differences in expertise, whether great or small, is crucial for offender assessment, treatment, and crime prevention (Nee, 2015). For instance, the extent that sexual burglary is an “expert” offense has important practical implications as sexual burglary can represent an important “stepping stone” to the development of a sexual criminal career (e.g., Schlesinger & Revitch, 1999) and may even be indicator for even more serious homicide offending (Vaughn et al., 2008). Conversely, empirical examination of sexual robbery as a distinct domain is extremely limited. As a result, there is little insight into variations in offense-related competencies within the sexual robbery domain, which may also shed light on primary motivations (i.e., sexual or theft). By examining two types of hybrid crimes, we, therefore, sought to contribute not just to the criminal expertise literature but also address the need for more criminological research that focuses on these complex crimes (Beauregard & Chopin, 2020). Accordingly, the present study seeks to explore latent subgroups of criminal expertise in both sexual burglary and sexual robbery.

## Literature Review

### Conceptualizing Criminal Expertise

The distinction between an “expert” in nonoffending domains and “criminal expertise” is important to note because the latter does not necessarily infer that an offender is “specialized” in their criminal career to be an expert. Specialization in criminal careers is generally referred to as the perceived probability of repeating the same type of crime when arrested next (Blumstein et al., 1986). Thus, “specialists” refer to individuals who specialize in one particular crime and engage in that behavior repeatedly and frequently (Simon, 1997). Conversely, the criminal expertise perspective acknowledges that some offenders will develop their expertise over time through practice, however, some offenders may also become “experts” even without continual practice (i.e., “dys” functional expertise; Nee & Ward, 2015). In other words, criminal expertise neither assumes offense “specialization” as a necessary component to the development of expertise nor does it require that an offender has an extensive previous history in their offense domain to develop expertise. Nonetheless, research findings indicate that for property offenders in particular, specialization increases with age (e.g., Armstrong, 2008; Nieuwbeerta et al., 2011). Thus, it seems that at least some level of specialization accrues with expertise, however, some expertise may be seen in nonspecialist offenders too, as a result of more confined practice (Nee et al., 2019). As such, criminal expertise should be considered a multi-faceted concept that involves both the acquisition of specific skills and knowledge achieved through many years of intensive practice and competent instruction (Ericsson, 2006; Ericsson & Charness, 1994; Ward, 1999) as well as domain-specific functional expertise requiring less deliberate practice and occurring over much shorter time periods (Ward, 1999; Ó Ciardha, 2015).

For instance, Ó Ciardha (2015) and Ward (1999) outline various ways expertise can develop indirectly in sexual offending, even without the commission of a contact sexual offense. These include covert modeling and rehearsal (e.g., sexual fantasies, observational learning via other offenders) or symbolic modeling (e.g., pornography or literature), observational learning (via other offenders—e.g., online forums, pedophile groups, etc.), or through an offender’s own experience with sexual abuse (e.g., physical or sexual abuse as a child; Ó Ciardha, 2015; Ward, 1999). Research on mental stimulation, for instance, has demonstrated the more a person mentally rehearses and thinks about how to perform an action, the more likely they are to actually act on it (e.g., Taylor & Pham, 1996). For example, MacCulloch et al. (1983) found that individuals with repetitive sadistic masturbatory fantasies can become compelled to seek out opportunities to “try-out” their fantasies, leading to increasingly more dangerous behavior. Thus, mental rehearsal is thought to provide an arena for offenders to plan, practice, and develop their expertise (Ward, 1999).

#### Rational choice theory and criminal expertise

On one hand, it has been argued that successfully engaging in criminality does not require special skills (Hirschi, 1986), but others have argued that this apparent “absence in decision-making” is not an indication of a lack of skills and planning, but rather, demonstrates that some offenders have developed in-depth knowledge and skills that allow them to make better and more instantaneous decisions, particularly in situations that require urgent action (Nee & Meenaghan, 2006). Thus, one of the key tenets of criminal expertise is that offenders who are experts will have domain-relevant knowledge stored in cognitive scripts, and once activated, these scripts enable them to process information and make decisions rapidly, resulting in improved performance (Nee et al., 2019; Ward, 1999). Criminal expertise can therefore be directly linked to rational choice theory (RCT), which suggests that a “bounded rationality” (Cornish & Clarke, 1986) in offender decision-making is related to the use of heuristics formed through prior learning, to maximize gain and minimize risk in their offending behavior. Since then, more refined versions of RCT have been developed that more directly address the involvement of automatic processes (Wortley, 2014) and the involvement of emotion (Van Gelder et al., 2013) in decision-making. Thus, individuals are thought to use an “adaptive toolbox” based on simple rules and heuristics to make most decisions, as opposed to a strict cost-benefit analysis more commonly associated with RCT (see Gigerenzer & Selten, 2002, for a review). Criminal expertise can also extend rational choice perspectives by enhancing our understanding of differences in offender decision-making by shifting the focus from social and psychological deficits to areas of competency and skill that facilitate decision-making (Fortune et al., 2015).

#### Continuum of expertise

Although there has been considerable variation in the application of the expertise framework to offending populations, one aspect that is widely agreed upon is that criminal expertise is best represented as a continuum novice to expert (e.g., Bourke et al., 2012; Chopin et al., 2021; Nee & Ward, 2015; Ward, 1999). In the field of sexual offending, a continuum of expertise has been observed in child sexual offending domains (Bourke et al., 2012) as well as adult rape (Chopin et al., 2021). These studies have found support for a novice to expert continuum, showing that more “expert” offenders possessed superior offense skills and knowledge, especially pertaining to detection avoidance, and gaining victim compliance and control (Bourke et al., 2012; Chopin et al., 2021). These studies, therefore, confirmed Ward’s (1999) assertions that experts in sexual offending would possess various offense related competencies and skills related to planning an offense, knowing how to avoid detection and how to respond to various contingencies, such as victim resistance (Ward, 1999). Moreover, according to Chopin et al. (2021), expert sexual offenders will be those who possess a sophisticated modus operandi in addition to “forensic awareness.”

Although the field of sexual offending has been slower to adopt the notion of a continuum of expertise, Nee and colleagues have made considerable advancements within the study of burglary. For instance, studies have shown support for a continuum of expertise between burglars and non-offenders (i.e., “novices”; Nee & Taylor, 2000; Nee et al., 2015; Nee & Taylor, 1988), as well as between burglars and non-burglar offenders (Logie et al., 1992; Nee et al., 2019). Taken together, these studies have found that burglars use distinctive and systematic routes, relied on previous learning when selecting a target, and have significantly better recognition memory and knowledge about burglary relevant cues compared with “novice” offenders and non-offenders. Although less attention has been given to differences between burglars, studies have also shown a novice to expert continuum within the burglary domain (Clare, 2011; Nee & Meenaghan, 2006). For instance, Nee and Meenaghan (2006) found that among 50 experienced residential burglars, two thirds showed evidence of expertise, but there was also a small number of more opportunistic offenders at the less experienced end, as well as more proficient and skilled searchers, and those who planned extensively at the end of the spectrum.

Considering that large body of expertise literature that suggest that burglary is an “expert” offense that involves substantial skill development, it is surprising that more attention has not been given to other types of offense that co-occur with burglary, such as sexual assault. For instance, Pedneault, Beauregard, et al. (2015) found that sexual motivated burglaries were not just a “bonus” to theft but were rationally oriented around situational cues that were markedly different from regular burglars (i.e., targeting occupied homes), suggesting they may possess a different domain-specific expertise. However, past studies on sexually motivated burglary (e.g., Pedneault et al., 2012, Pednault, Beauregard, et al., 2015) have included non-contact “fetish” break-ins. Thus, the heterogeneity of contact sexual burglary (i.e., a “hybrid” offense, involving break-and-entering, theft, and sexual assault) and its varying degrees of skill and offense-related competencies has never been fully examined. Nonetheless, variations in the degree of expertise are especially important to examine with this population, given that sexually motivated burglaries are associated with sexual dangerousness (Schlesinger & Revitch, 1999; Vaughn et al., 2008), escalation in offending (Harris et al., 2013; Pedneault, Harris, & Knight, 2015), and even sexual homicide (e.g., Schlesinger & Revitch, 1999).

### Criminal Expertise and Hybrid Offenses

A hybrid offense refers to a crime that has been produced by the combination of two or more distinct offense elements (Beauregard & Chopin, 2020). As such, the term “hybrid” reflects the nature of the offense—not necessarily the offender’s motivations. Sexual burglary is a distinct type of “hybrid offense,” that for the purposes of the current study, is defined as the act of unlawful entry into a building in addition to personal theft of property and contact sexual assault. Similarly, sexual robbery, is a distinct type of “hybrid offense” that for the purposes of this study, is defined as sexual assault in addition to personal theft of property by force from a victim. Our previous study (Reale et al., in press) was the first to apply the expertise framework to hybrid offending to determine differences in criminal expertise between these two subgroups. We hypothesized that sexual burglary may be a more “expert” offense compared with sexual robbery, given that burglary is thought to involve substantial skill development and greater expertise (e.g., Nee, 2015).

Interestingly, we found that the crime-commission process of sexual burglary involved domain-specific expertise because of the skilled behavior across the pre-crime, crime, and post-crime phases (e.g., protecting their identity) and superior detection avoidance skills (e.g., destroying and removing evidence) relevant to the high-risk nature of the offense. However, both domains also possessed skills in the pre-crime phase (e.g., victim and location selection), suggesting some important similarities in expertise (i.e., “overlapping expertise”; Nee et al., 2019) related to the interpersonal nature (i.e., high level of victim-offender interaction) of these offenses as well (Reale et al., in press). Nonetheless, we did not account for whether criminal expertise varies (i.e., on a continuum from novice to expert) within each of these domains (i.e., sexual burglary and sexual robbery). Thus, it remains unclear whether there are “novice” types in sexual burglary who may have less skills developed, or whether sexual burglary comprises of a large group of skilled offenders or “experts,” as observed in burglary (e.g., Nee & Meenaghan, 2006).

In comparison to sexual burglary, sexual robbery has received much less empirical attention and the extent that these offenses are “bonuses” to theft or sexually motivated is currently unknown. In our previous study (Reale et al. in press), we observed that sexual robbery involved similar offense related competencies (e.g., planning of targets and victim compliance methods) that have been found among more experienced robbery offenders (Deakin et al., 2007; Smith, 2003) and opportunistic sexual offenders (Rossmo, 2000). Nonetheless, we did not examine whether there is a subgroup of “experts” in sexual robbery who share similar skills in detection avoidance that has been observed in other “experts” in rapei (Chopin et al., 2021) and sexual burglary (Reale et al., in press). Thus, an examination of the heterogeneity of criminal expertise in both sexual robbery and burglary may reveal important differences in underlying decision-making processes between novice to expert offenders, but also shed light onto differences in primary motivations, which is crucial for accurate offender assessment, intervention, and rehabilitation (Bourke et al., 2012; Fortune et al., 2015; Nee et al., 2019; Ward, 1999).

## Current Study

Although there has been considerable variation in the application of expertise to offending populations, one aspect that is widely agreed upon is that expertise is best represented on a continuum from novice to expert (Bourke et al., 2012; Nee & Ward, 2015; Reale et al., in press; Ward, 1999). The current study, therefore, aims to build upon our previous study (Reale et al., in press) by exploring differences in latent subgroups of criminal expertise (i.e., between novices to expert) for each hybrid sexual burglary and sexual robbery domain. We focus on capturing criminal expertise through the crime-commission process because it provides an objective and systematic way to measure behavioral indicators of expertise that allows for comparisons between studies. We therefore use the expertise literature (e.g., Bourke et al., 2012; Nee & Ward, 2015; O̕ Ciardha, 2015; Ward, 1999) as well as empirical studies on skilled decision-making and criminal sophistication in sexual offending (e.g., Beauregard & Proulx, 2017; Chopin et al., 2019; Chopin et al., 2021; Davies et al., 1997; Park et al., 2008; Reale et al., in press), burglary (e.g., Nee, 2015; Nee & Meenaghan, 2006; Nee & Taylor, 2000) and robbery (e.g., Deakin et al., 2007; Wright & Decker, 2002) to formulate behavioral indicators of expertise for the current study.

## Method

### Sample

This study is based on a sample of 877 solved (i.e., a suspect has been charged and apprehended by police) hybrid stranger sexual assault and forcible theft/burglary cases against female victims that occurred in France between 1992 and 2018.^1^ Cases included in the present study must include a sexual assault (i.e., a contact sexual offense) and involve either a (a) burglary (break-and-enter + theft) or (b) robbery (forcible theft only).^2^ All cases are single-incident sexual offenses (i.e., there are no detected serial sexual offenders in the sample). We also chose to focus on stranger sexual assaults, not only because these cases tend to be more difficult for police to solve (e.g., Bouffard, 2000) but also because acquaintance rapes have been found to have distinctive offending patterns from stranger rapes (see Bownes et al., 1991; Koss et al., 1988).

Finally, we chose to retain offenses that occurred during the 1992–2000s (14.4%). This is important to note as some variables included in the study relate to strategies to avoid detection (e.g., destroying and removing evidence, or wearing gloves) that are associated with “forensic awareness” (Davies, 1992). Although the use of DNA evidence emerged in the 1980s, it was not widely used by French Police until 1998 (Vailly & Bouagga, 2019), and as a result, some concerns with capturing “forensic awareness” variables in offenses that occurred during the 1990s are warranted. This possibility is limited, however, given the fact that a large proportion of the cases (86.6%) occurred since the year 2000. Moreover, we do not find any significant statistical association related to the date of the offense occurring more recently and the use of forensic awareness strategies (i.e., protecting identity and destroying/removing evidence).^3^ It is also important to consider that the influence of DNA evidence on offender behavior was accounted for in studies even before the emergence of national police DNA databases (e.g., Davies, 1992; Davies et al., 1997). In addition, Beauregard and Martineau (2015) found in their sample of sexual homicide, that the use of forensic awareness strategies was stable (*M* = 0.4–0.5) across 1991–2010. As such, we do not believe this constitutes as a methodological concern in the present study.

The sample was obtained from a national police database operated by the Ministry of Interior in France. Crime analysts maintain this database by using different sources of information (e.g., forensic and investigative reports, witness and offender interviews, etc.) related to the criminal case. Detailed and unique information about the crime-commission process (e.g., whether a victim was targeted, whether an offender selected a familiar or deserted location) is completed by criminal investigators assigned to the case and is recorded in investigative files that are compiled, analyzed and entered into the database by a team of crime analysts who are experts in violent crimes. Information related to forensic awareness strategies, forensic services, legal medicine, and interviews with the victims and offenders, which are then compiled and entered into this database. Although missing data are possible, for the current study, there are no missing data for any of the variables used. Ethical approval was obtained for this research.

### Analytical Strategy

Our analytical strategy included a two-step process. First, to determine the extent that criminal expertise behaviors vary for both sexual burglary and sexual robbery, we employed latent class analysis (LCA) using Latent Gold V5.1 software package. LCA is a statistical procedure used to identify heterogeneity that is not directly observable or measurable and therefore can be used to detect patterns in a set of data or subgroups based on shared behavioral characteristics (Collins & Lanza, 2010). More specifically, LCA is to identify mutually exclusive cases (i.e., with no overlap) on the basis of dichotomous indicator variables (Lanza et al., 2007, 2003). LCA is similar to cluster analysis but provides stronger models because it attributes class membership probabilities to each individual case. We computed and analyzed one-to-seven class solutions for both samples separately (see Tables 1 and 2). The Bayesian information criterion (BIC) was used to evaluate the model fit and determine the number of classes to use in LCA. Schwarz (1978) mentioned that a lower BIC value indicates an improvement in the fit of models. We also used other fit measure: log-likelihood ratio L^2^, degrees of freedom, Akaike information criterion (AIC), and entropy. In the second step, we used additional variables to improve the depth of our models. We used bivariate analysis (i.e., chi-square analysis and Kruskal–Wallis Test) and post hoc testing to identify significant differences between the subgroups.

**Table 1.** Fit Indices for Latent Classes: Sexual Burglary.

Nb of classes	Log likelihood	L^2^	*Df*	BIC	AIC	Entropy
1	−2,599.75	1,178.38	380	5,221.50	5,265.16	1.00
2	−2,414.66	808.19	368	4,966.59	4,875.31	0.91
3	−2,362.58	704.04	356	4,934.07	4,795.16	0.83
**4**	−**2,326.13**	**631.13**	**344**	**4,932.78**	**4,746.26**	**0.81**
5	−2,309.70	598.28	332	4,971.55	4,737.40	0.82
6	−2,297.73	574.34	320	5,019.24	4,737.46	0.84
7	−2,289.29	557.45	308	5,073.97	4,744.57	0.81

*Note.* BIC = Bayesian information criterion; AIC = Akaike information criterion.

**Table 2.** Fit Indices for Latent Classes: Sexual Robbery.

Nb of classes	Log likelihood	*L* ^2^	*df*	BIC	AIC	Entropy
1	−2,979.62	1,297.14	467	6,027.12	5,981.25	1.00
2	−2,691.62	721.12	455	5,525.14	5,429.24	0.96
3	−2,635.07	608.02	443	5,486.08	5,340.14	0.89
**4**	**−2,597.08**	**532.04**	**431**	**5,484.13**	**5,288.16**	**0.87**
5	−2,580.33	598.53	419	5,524.67	5,278.66	0.86
6	−2,572.25	482.38	407	5,582.55	5,286.51	0.88
7	−1,972.75	464.25	395	5,638.44	5,292.37	0.84

*Note.* BIC = Bayesian information criterion; AIC = Akaike information criterion.

### Indicator Variables

On the basis of previous studies, we selected 11 dichotomous variables (0 = no, 1 = yes) related to criminal expertise and sophisticated modus operandi in sexual offending to identify underlying patterns or subgroups of individuals (Bourke et al., 2012; Chopin et al., 2019; Chopin et al., 2021; Park et al., 2008; Reale et al., in press; Ward, 1999). Apart from (a) whether the offender has a previous criminal history (i.e., had a previous charge or conviction), these behaviors have been grouped into six behavioral themes directly related to the crime-commission process. The *Planning* theme includes, (b) victim was targeted (i.e., selected based on specific characteristics); (c) offender brought a weapon to the offense. The *Precautions* theme includes, (d) offender acted on the environment (i.e., offender took precautions specific to their surroundings, such as disabled the alarm/phone, blocked exits, etc.); (e) acted on victim (i.e., took precautions specific to the victim, such as blindfolding or gagging the victim, threatening not to report, using restraints, etc.); (f) protected identity (e.g., wore a mask, disguise, or gloves). The *Sexual Acts* theme includes (g) vaginal/anal intercourse. The *Violence* theme includes (h) non-sexual manual violence (e.g., beating, choking, crushing); (i) weapon used (e.g., knife, gun, blunt object). The *Control* theme includes (j) victim was intentionally released by offender. Finally, the *Forensic Awareness* theme includes (k) destroyed or removed forensic evidence.

### Additional Variables

To provide a more comprehensive understanding of the different subgroups of criminal expertise, as well as to provide external validity to our subgroups, we examined additional variables focused on victim, offender, and location characteristics.

#### Victim characteristics

Victim characteristics included one continuous variable: (a) age of victim (*M* = 35.04, *SD* = 18.38, range = 14–94) and two dichotomous variables (0 = no, 1 = yes); (b) single; and (c) used drugs or alcohol prior to crime. Studies have shown that more criminally sophisticated offenders tend to target their victims, especially those who are vulnerable (e.g., Chopin et al., 2021; Beauregard & Proulx, 2017; Wright & Decker, 2002).

#### Offender characteristics

Offender characteristics included one continuous variable: (a) age of offender (*M* = 28.59, *SD* = 8.43, range = 16–71) and four dichotomous variables (0 = no, 1 = yes): (b) married/common-law; (c) has a sexual dysfunction; (d) has paraphilic behaviors; (e) possessed apornography collection. Variables 1 and 2 were based on previous studies that suggest criminally sophisticated sexual offenders will be older and socially adept (e.g., Bourke et al., 2012; Ward, 1999). Variables 3–5 were included to provide greater insight into the offender’s sexual history and the role this may play in the development of skilled behaviors during the crime-commission process. For instance, studies have suggested that different mechanisms (e.g., sexual fantasies; pornography consumption) allow for the development of offense-related skills and knowledge as they can serve as way to practice, plan, or mentally rehearse offenses (Ó Ciardha, 2015; Ward, 1999).

#### Location characteristics

Location included two dichotomous variables (0 = no, 1 = yes): (a) location was familiar to offender and (b) offender selected a deserted location (i.e., where witnesses are unlikely to hear, see, or interrupt the crime). These variables were included based on previous studies, which have suggested that offenders with greater expertise tend to target locations where there is a lower risk of detection and that are familiar to them to enable quicker getaways (Nee, 2015).

## Results

A four-class solution provided the best overall fit for both the sexual burglary and sexual robbery data (see Tables 1 and 2). The BIC (Schwarz, 1978) is a penalized log-likelihood model information criterion that can be used to compare competing model fit to the same data (i.e., models with different numbers of latent classes). For the sexual burglars and robbers, the BIC decreases up to four classes, and the addition of more classes provides no improvement to model fit. AIC values decreased slightly after Model 4 for both samples, however, parsimony was favored to improve interpretability of the models. Moreover, the final four class models selected for both samples presented high classification accuracy (entropy) based on posterior probabilities, confirming its stability and relevance. Tables 3 and 4 show, for each latent subgroup, the assigned probability of membership as well as the item–response probabilities for each subgroup. The item–response probabilities vary from 0 to 1.00; item–response probabilities closer to 1.00 indicate the presence of the item for the class. Item–response probabilities falling between .45 and .63 were interpreted as somewhat arbitrary presence of the items (Deslauriers-Varin & Beauregard, 2010). Additional bivariate analyses were computed for dichotomous (χ^2^ test) and continuous (Krusal–Wallis test) variables, to test for differences between latent class subgroups for both sexual burglary (Table 5) and sexual robbery (Table 6). Finally, post hoc testing was conducted using ɀ tests to compare subgroups for statistically significant differences (see Tables 5 and 6). Bonferroni correction method was used for each row to control for Type 1 error (Sharpe, 2015).

**Table 3.** Profile of Four Latent Sexual Burglary Classes: Mean Probabilities of Criminal Expertise Characteristics Based on Class Membership.

Criminal expertise	Indicator variables	Sexual burglary				
		Novice	Intermediate	Intermediate	Expert	
		Class 1	Class 2	Class 3	Class 4	Overall
		0.31	0.28	0.22	0.19	1.00
		*N* = 115	*N* = 121	*N* = 87	*N* = 68	391
1. Criminal history	Previous convictions	0.19	0.23	0.35	0.27	0.25
2. Planning	Victim targeted	0.19	0.23	0.21	0.40	0.25
	Weapon brought	0.01	0.00	0.88	0.83	0.36
3. Precautions	Acted on environment	0.00	0.46	0.00	0.80	0.28
	Acted on victim	0.22	0.99	0.62	0.99	0.68
	Protected identity	0.21	0.40	0.37	0.68	0.39
4. Sexual acts	Vaginal/Anal intercourse	0.51	0.77	0.69	0.75	0.67
5. Violence	Nonsexual (manual) violence	0.30	0.42	0.30	0.18	0.31
	Weapon used	0.05	0.19	0.99	0.99	0.48
6. Control	Victim intentionally released	0.63	0.87	0.79	0.87	0.78
7. Forensic Awarness	Destroyed or removed evidence	0.06	0.23	0.12	0.47	0.20

**Table 4.** Profile of Four Latent Sexual Robbery Classes: Mean Probabilities of Criminal Expertise Characteristics Based on Class Membership.

Criminal expertise	Indicator variables	Sexual robbery				
		Novice	Intermediate	Intermediate	Expert	Overall
		Class 1	Class 2	Class 3	Class 4	
		0.39	0.16	0.22	0.23	1.00
		*N* = 181	*N* = 82	*N* = 91	*N* = 124	478
1. Criminal history	Previous convictions	0.20	0.13	0.19	0.26	0.20
2. Planning	Victim targeted	0.12	0.16	0.19	0.21	0.16
	Weapon brought	0.00	0.00	0.94	0.99	0.44
3. Precautions	Acted on environment	0.00	0.53	0.00	0.35	0.17
	Acted on victim	0.19	0.99	0.14	0.99	0.50
	Protecting identity	0.18	0.27	0.41	0.51	0.32
4. Sexual acts	Vaginal/anal intercourse	0.49	0.75	0.57	0.75	0.61
5. Violence	Nonsexual (manual) violence	0.31	0.41	0.30	0.22	0.30
	Weapon used	0.01	0.21	0.99	0.99	0.49
6. Control	Victim intentionally released	0.65	0.75	0.67	0.85	0.72
7. FAS	Destroyed or removed evidence	0.02	0.11	0.02	0.17	0.07

**Table 5.** Bivariate Associations Against Sexual Burglary Latent Classes.

	Sexual Burglary				Test statistic
	Class 1	Class 2	Class 3	Class 4	
	29.4%	30.9%	22.3%	17.4%	
	115	121	87	68	
Additional Variables	*N* (%)	*N* (%)	*N* (%)	*N* (%)	
Offender characteristics					
Age^a, b^	29.1 (9.9)	28.0 (6.9)	27.0 (7.1)^c^	30.8 (9.3)^d^	7.68^†^
Married/common law	17 (14.8)	15 (12.4)	20 (23.0)	8 (11.8)	5.42
Any sexual dysfunction	12 (10.4)^c^	26 (21.5)^c^	15 (17.2)^c^	29 (42.6)^d, e,f^	27.73 ***
Any paraphilias	25 (21.7)	18 (14.9)	12 (14.9)	16 (23.5)	3.74
Pornography collection	6 (5.2)^c^	5 (4.1)^c^	4 (4.6)^c^	18 (26.5)^d, e,f^	34.72 ***
Victim characteristics					
Age^a, b^	37.7 (19.4)^d^	37.6 (20.8)^d^	30.2 (12.8)^e, f^	32.2 (16.5)	6.53^†^
Single/unmarried	29 (25.2)	44 (36.4)	33 (37.9)	27 (39.7)	5.84
Used drugs/alcohol prior to offense^g^	23 (20.0)^c, d^	14 (11.6)	5 (5.7)^e^	3 (4.4)^e^	14.34 **
Location					
Location was familiar to offender	31 (27.0)	26 (21.5)	15 (17.2)	18 (26.5)	3.26
Offender selected a deserted location	55 (47.8)^c^	72 (59.5)	56 (64.4)	49 (72.1)^e^	11.79 **

a *Mean* (*SD*). ^b^ Kruskal–Wallis Test. ^c^ Significant difference with Class 4. ^d^ Significant difference with Class 3. ^e^ Indicates significant difference with Class 1. ^f^ Significant difference with Class 2. ^g^ Fisher’s Exact Test.

† *p* = .10. *p ≤ .05. **p < .01. ***p <.001.

**Table 6.** Bivariate Associations Against Sexual Robbery Latent Classes.

	Sexual Robbery				Test Statistic
	Class 1	Class 2	Class 3	Class 4	
	37.8%	26.1%	19.0%	17.1%	
	181	125	91	82	
Additional Variables	*N* (%)	*N* (%)	*N* (%)	*N* (%)	
Offender characteristics					
Age^a, b^	28.7 (8.9)	28.6 (7.8)	28.4 (7.8)	28.29 (7.3)	0.05
Married/common law	38 (21.0)	24 (20.0)	18 (19.8)	16 (19.5)	0.16
Any sexual dysfunction	9 (5.0)^c, d,e^	27 (21.8)^c^	17 (18.7)^c^	15 (18.3)^c^	21.01 ***
Any paraphilias	30 (16.6)	21 (16.9)	17 (18.7)	15 (18.3)	0.13
pornography collection^f^	7 (3.9)	12 (9.7)	8 (8.8)	3 (3.7)	6.16
Victim characteristics					
Age^a, b^	28.9 (13.7)	27.9 (11.3)	27.3 (11.8)	27.8 (12.2)	0.44
Single/unmarried	68 (37.6)	48 (38.7)	38 (41.8)	25 (30.5)	2.50
Used drugs/alcohol prior to offense^f^	28 (15.5)^d^	4 (3.2)^c^	5 (5.5)	10 (12.2)	15.04 ***
Location					
Location was familiar to offender	88 (48.6)	51 (41.1)	49 (53.8)	30 (36.6)	6.85^†^
Offender selected a deserted location	87 (48.1)^d, g^	93 (75.0)^c^	59 (64.8)	54 (65.9)^c^	24.38 ***

a *Mean* (*SD*). ^b^ Kruskal–Wallis Test/F statistic. ^c^ Indicates significant difference with Class 1. ^d^ Significant difference with Class 2. ^e^ Significant difference with Class 3. ^f^ Fisher’s Exact Test. ^g^ Significant difference with Class 4.

† *p* = .10. *p ≤ .05. **p < .01. ***p <.001.

### Latent Criminal Expertise Subgroups

#### Novices

Sexual Burglary—Class 1 (29.4%). This subgroup was classified as “novice” due to the lack of planning, precautions, and sexually intrusive acts associated with this group (see Table 3 for details). This subgroup also had the lowest likelihood of previous convictions (0.19) compared with the other sexual burglary subgroups. They were also characterized by never bringing a weapon to the offense (0.05), and they were the least likely subgroup to have intentionally released their victim (0.63) or to have destroyed or removed evidence (0.06). This subgroup was also the youngest (*M* = 27.0, *SD* = 7.1) relative to other subgroups, although this association was only approaching significance, χ^2^ (3) = 7.68, *p* = .053. In addition, bivariate analysis (see Table 5) indicated that this subgroup was most likely to have victims who used drugs or alcohol prior to the offense (20.0%) compared with Class 3 and 4, χ^2^ (3) = 14.34 *p* = .002, φ = .192. Offenders in this subgroup also selected a deserted location at the lowest frequency (47.8%), compared with class 4, χ^2^ (3) = 11.79, *p* = .008, φ = .174.

#### Novices

Sexual Robbery—Class 1 (37.9%). This is the largest subgroup observed among sexual robbery and is classified as “novice” due to the lack of planning, precautions, and sexually intrusive acts associated with this group (see Table 4 for details). They were also characterized by never bringing a weapon to the offense (0.00), and they are the least likely subgroup to have intentionally released their victim (0.65) and never destroyed or removed evidence (0.02). This subgroup also has a low likelihood of previous convictions (.20) although this is comparable to the average odds for the sample of sexual robbers (0.20). Bivariate analysis (see Table 5) indicated that offenders in this subgroup were significantly less likely to have a sexual dysfunction (5.0%) compared with other classes, χ^2^ (3) = 21.01, *p* <.001, φ = .210. This subgroup was most likely to have victim who used drugs or alcohol prior to the offense (15.5%), compared with Class 2, χ^2^ (3) = 15.04 *p* = .002, φ = .177. Offenders in this subgroup selected a deserted location at the lowest frequency (48.1%) compared with Class 3 and 4, χ^2^ (3) = 24.37, *p* =<.001, φ = .226.

#### Intermediate–manual violence

Sexual Robbery—Class 2 (17.2%). This represents the smallest subgroup in sexual robbery and is characterized as “intermediate” due to their precautions taken related to acting on the victim (0.99) and the high likelihood of sexually intrusive acts (0.75). This subgroup is labeled as “manual violence” because they never brought a weapon to the offense (0.00) but had the highest odds of nonsexual violence against the victim (0.41). The likelihood of offenders in this subgroup to have previous convictions (0.13) was the lowest among subgroups (see Table 4 for more details).

#### Intermediate—weapon related

Sexual Robbery—Class 3 (19.0%). This subgroup is characterized as “intermediate- weapon related” due to the weapon use and some planning/precautions (see Table 4 for more details). This group lacked precautions (see Table 4 for details), but sometimes protect their identity (0.41). They are very likely to have brought a weapon (0.94) and to have used it (0.99) during their crime. Sexually intrusive acts only sometimes occurred (0.57), and they never destroyed or removed evidence (0.02).

#### Intermediate–manual violence

Sexual Burglary—Class 2 (30.9%). This is the largest subgroup for sexual burglary and is characterized as “intermediate” due to their precautions related to acting on their victim (0.99) and the high likelihood of sexually intrusive acts (0.770. This subgroup was labeled as “manual violence” because they never brought a weapon to the offense (0.00) and the highest odds of non-sexual violence (0.42), relative to other subgroups. This subgroup was very likely to intentionally release their victim (0.87) and although unlikely, they destroy or remove evidence (0.23) at the second-highest rate among sexual burglary subgroups (see Table 3 for more details). The likelihood of offenders in this subgroup to have previous convictions (0.23) was lower than both Classes 3 and 4 (see Table 3 for more details).

#### Intermediate—weapon related

Sexual Burglary—Class 3 (22.3%). This subgroup was characterized as “intermediate- weapon related” by their weapon use and some planning and precautions (see Table 3 for more details). This subgroup was the most likely among sexual burglary to have a history of previous convictions (0.35). They were very likely to have brought a weapon (0.88) and to have used it (0.99) during their crime. They were likely to commit sexually intrusive acts (0.69) although this is the lowest odds for all sexual burglary subgroups. They were also unlikely to have destroyed or removed evidence (0.12).

#### Expert

Sexual Robbery—Class 4 (25.9%). This subgroup is labeled as “expert” as they were the most likely subgroup in sexual robbery to take precautions (see Table 4 for details) and always brought a weapon with them to their offense (0.99). This group was the most likely to commit sexually intrusive acts (0.75) and to intentionally release their victim (0.85). Finally, although odds remain low, this subgroup was the most likely among sexual robbery to have destroyed or removed evidence (0.17). This subgroup is also the most likely to have a history of previous convictions (0.26) compared with other subgroups. This subgroup is also associated with offenders who selected deserted locations (75.0%), compared with the “novice” sexual robbery subgroup (class 1), χ^2^ (3) = 24.37, *p* =<.001, φ = .226.

#### Expert

Sexual Burglary—Class 4 (17.4%). This was the smallest subgroup in sexual burglary but is characterized as “expert” as they are the most likely subgroup to show evidence of expertise across the pre-crime, crime, and post-crime phases. Planning is evident in that they have a high probability of having taken all forms of precautions (see Table 3 for details). They were likely to have engaged in intrusive sexual acts (0.75) but unlikely to have used non-sexual violence (0.18). This subgroup was most likely to intentionally release their victims (0.87) and to destroy and remove evidence (0.47). This subgroup was the second most likely to have a history of previous convictions (0.27) compared with other sexual burglary subgroups. In terms of bivariate findings, offenders in class 4 were the oldest (*M =* 30.8, *SD* = 9.3), although this association is only approaching significance: χ^2^ (3) = 7.68, *p* = .053. Offenders in this subgroup were also associated with having a sexual dysfunction (42.6%) compared with the other classes, χ^2^ (3) = 27.73, *p* = <.001, φ = .226, and a pornography collection (26.5%) compared with other classes, χ^2^ (3) = 34.72, *p* = <.001, φ = .298. This subgroup was also associated with offenders who selected a deserted crime location (72.1%) compared with Class 1—novice, χ^2^ (3) = 11.79, *p* = .008, φ = .174.

## Discussion

The current study examined latent subgroups of criminal expertise in both sexual burglary and sexual robbery. Our findings supported existing research indicating that criminal expertise varies from novice to expert between offenders within the same domain (Bourke et al., 2012; Chopin et al., 2021; Clare, 2011; Nee & Meenaghan, 2006). More specifically, we identified an “expert” subgroup for both domains whose offense-related competencies were distinctive from “novice” and “intermediate” subgroups. By examining the heterogeneity of criminal expertise in both samples, we were also able to expand on our previous findings (Reale et al., in press) to offer more insight into differences into motivation of these hybrid offenses (i.e., primarily sexual or theft-related motives) as well as highlight the differences in decision-making along the expertise continuum (i.e., from novice to expert). Moreover, because we examined two hybrid offending domains that shared overlapping characteristics (i.e., theft and sexual offense elements), this allowed us to determine whether there was “transferrable expertise” (Nee et al., 2019) related to the interpersonal violent nature of these crimes (i.e., a high level of victim-offender interaction) as well as whether there are domain-specific “experts” in sexual burglary and sexual robbery. We discuss each of the latent criminal expertise subgroups and their theoretical and practical implications in the following subsections.

### Continuum of Expertise

#### Novices

We found a novice subgroup for both sexual burglars (29.4%) and sexual robbers (37.8%) that were best characterized by their lack of skill, planning, and the absence of sophisticated behaviors over the entire crime-commission process. The fact that novices also did not a bring weapon to their offense suggests a lack of planning and preparedness for an offense that requires a high level of victim-offender interaction. Thus, it appears that offenders in the novice subgroups were either not concerned with, or did not yet possess the necessary skills, to reduce their risk of apprehension.

These findings are similar to the novice rapist found by Chopin et al. (2021), who were described as having a basic modus operandi and the absence of forensic awareness. For both sexual burglary and sexual robbery, novice subgroups were the least likely to have selected a deserted crime scene location (where there is less risk of being seen or heard) or target their victims, suggesting that they lack target appraisal skills that are found in more experienced burglary (e.g., Nee & Meenaghan, 2006; Wright et al., 1995) and robbery offenders (e.g., Deakin et al., 2007; Wright & Decker, 2002) and even sexual offenders (e.g., Rossmo, 2000). Moreover, victims of novice subgroups were more likely to be under the influence of alcohol, suggesting that “novice” offenders simply acted on an opportunity to victimize a vulnerable victim with little thought to the long-term consequences (i.e., apprehension). As a whole, it is the novice subgroup that closely aligns with the assertion made by Hirschi (1986) that offenders are “not very good at what they do” (pg. 115-116) with their novice skill set, these findings suggest novice offenders may lack experience in both domains (i.e., sexual offending and/or burglary/robbery).

### Intermediates

#### Sexual Robbery subgroup (19.0%)—weapon related

Offenders in this subgroup always brought and used a weapon but only sometimes took precautions to protect their identity and they never destroyed or removed evidence. As such, it is possible that these offenders were in a “state of readiness” (Nee, 2015)—or exhibited “premeditated opportunism” (Rossmo, 2000)—for a violent encounter and chose to both sexually assault and steal from their victim because the conditions allowed for both with little increased risk. Taken together with the lower likelihood of sexually intrusive acts occurring (relative to other subgroups) and the lack of precautions taken to control the victim, this suggests that robbery may have been the primary motivation and the sexual assault occurred as an afterthought or “bonus” to the robbery.

#### Sexual Burglary subgroup (22.3%)—weapon related

Offenders in this subgroup always brought and used a weapon, suggesting that they are expecting to come into contact with a victim during the burglary, yet they only sometimes take precautions to reduce the risks associated with interpersonal offending. This represents an interesting subgroup of sexual burglary because weapon use among sexual offenders is typically rare (Beauregard & Leclerc, 2007). Considering that this subgroup were the most likely among sexual burglary subgroups to have a history of previous conviction, these findings may be indicative of previous experience with violent offending. For instance, weapon use in sexual burglary has been linked with more serious and violent criminal careers (DeLisi et al., 2017; Vaughn et al., 2008). Lastly, this subgroup is consistent with the “versatile” contact sexual burglaries subgroup found in Pedneault et al. (2012), which were similarly characterized by theft, violence, and weapon-use.

#### Sexual Robbery subgroup (17.2%)—manual violence

This is the smallest sexual robbery subgroup, who are best characterized by their manual acts of violence and precautions related to acting on the victim (e.g., blindfolding, gagging), presumably as a way to control their victim. The fact that they never brought or used a weapon during their offense is an important finding as this contrasts the “typical” robbery offender with theft motivations (e.g., Smith, 2003). Beauregard and Leclerc (2007) have shown that only a minority of sexual offenders use of a weapon to commit their crime, because it is either not necessary, the crime was not planned, or the use a weapon was not compatible with their fantasies. Thus, it is possible that this subgroup’s primary motivation was sexual, and the theft occurred out of opportunity. For instance, this group bears similarities to the “angry rapist,” whose goal is to express contempt for their victim through physical violence and typically acts to overpower the victim and achieve penetration (Groth, 1979).

#### Sexual Burglary subgroup (30.9%)—manual violence

This subgroup is the largest in our sample of sexual burglary. Offenders in this subgroup are best characterized by their skilled behaviors, particularly related to maintaining control of their victim as well as a greater likelihood of both nonviolent and sexually intrusive acts (i.e., vaginal/anal penetration). Offenders in this subgroup do not engage in a high level of planning (i.e., never bring a weapon and rarely target victims) but always take precautions that are specific to acting on the victim (e.g., blindfolding/gagging the victim). Outside of the expert sexual burglary subgroup, this subgroup is the most likely to protect their identity and act on their environment. Thus, it appears that offenders in this subgroup were fully expecting to break-and-enter a location where a victim was present. As such, offenders in this subgroup were highly likely to be sexually motivated and took the opportunity to steal from the victim because of opportunity, which is consistent with sexually motivated burglaries found in Pedneault, Beauregard, et al. (2015).

### Experts

#### Sexual robbery (25.9%)

This subgroup is labeled as sexual robbery “experts” as they are distinctive from both novices and intermediate sexual robbery subgroups in terms of offense-related competencies over the crime-commission process. More specifically, this subgroup is best characterized by violence related to weapon-use, and skilled behavior needed for the commission of a violent offense, including the ability to maintain control of the victim and selecting a low-risk location. Interestingly, skills related to the pre-crime phase (i.e., victim and location selection) have been observed in burglary offenders (see Nee, 2015 for a review), sexual offenders (Rossmo, 2000), and experienced street robbers (e.g., Deakin et al., 2007; Smith, 2003; Wright & Decker, 2002), suggesting a type of “overlapping” expertise (Nee et al., 2019). Thus, although these offenders may be “experts” in sexual robbery relative to novice and intermediates, they do not appear to have “domain-specific” expertise related to sexual offending. In other words, they do not possess the same level of detection avoidance competencies found in adult rape (Chopin et al., 2021), serial sex offenders (Park et al., 2008) or the “expert” sexual burglary offenders in the current study. Rather, they appear to have expertise in violent offending—this allows their skills to “overlap” or “transfer” across domains—enabling them to offend with “dys-functional expertise” in domains that involve a high degree of victim-offender interaction (i.e., violent offending domains).

#### Sexual burglary (17.4%)

This subgroup, although the smallest, represents the most important group in our model because this subgroup is most closely aligned with conceptualizations of an “expert” offender who possesses “domain-specific” or “specialized” skills and knowledge in their domain. According to Ward (1999), an expert in sex offending will strategize for how to select a victim, how to plan and successfully carry out an offense, how to avoid detection, and how to respond to various contingencies, such as victim resistance. We find support for these behavioral manifestations of expertise across the crime-commission process for this sexual burglary subgroup. Moreover, our experts in sexual burglary closely resemble the findings of Chopin et al. (2021) who found that “experts” in adult rape had both a sophisticated modus operandi and the use of specific strategies to increase their odds of eluding police detection. In particular, the act of destroying and removing evidence is associated with a more experienced and sophisticated sexual offender (Park et al., 2008) and was most likely among the “expert” sexual burglary subgroup. Taken with other behaviors indicative of an offender who is expected to encounter a victim, this suggests that “experts” in sexual burglary are likely to be sexually motivated and thus committed theft as a “bonus” to the burglary (Pedneault, Beauregard, et al., 2015).

In addition, several findings help to shed light on the development of expertise in this subgroup. Although odds of prior convictions were relatively low (although second most among sexual burglary subgroups) these offenders were considerably older than novice sexual burglary offenders. According to Ward (1999), expert offenders will be older, as they will have learned strategies to avoid detection over the course of their criminal career. Thus, the presence of detection avoidance skills provides an indication that these offenders have learned from prior offense experience and may have previous undetected sexual offenses (Ward, 1999). For instance, the fact that our sample of “expert” sexual burglary offenders all stole items from their victim may also be an indication that those who become “experts” in sexual burglary may start with burglary, or even fetish break-ins. Indeed, burglary has been suggested as a “gateway” toward sexual offending, and studies have found a link between burglary and escalation in dangerous offending (e.g., DeLisi et al., 2017; Harris et al., 2013; Pedneault, Harris, & Knight, 2015).

Interestingly, we also find that offenders in this subgroup are also the most likely to possess a pornography collection and have a sexual dysfunction. Frequent masturbation to deviant sexual fantasies has been argued to provide a form of emotional reinforcement and practice through mental rehearsal (Nee & Ward, 2015). This is consistent with Bourke et al. (2012), which found that this allowed expert child sexual offenders to refine their modus operandi tactics before implementing them. Specifically, reinforcement of sexual fantasies through masturbation was associated with heightened sexual desire in general, and also strengthened desires for specific victims as well (Bourke et al., 2012). As such, the sexual burglary subgroup may have obtained “specialist” knowledge and skills relevant to sexual offending as a function of both direct and indirect learning and experience.

### Theoretical Implications

By examining differences in the heterogeneity of expertise in both sexual burglary and sexual robbery, the current study revealed important differences in decision-making processes between “novice” and “expert” subgroups. In other words, we can extend rational choice perspectives by providing a better understanding of the psychological mechanism involved in the commission of a crime (Nee et al., 2019). For instance, dual-systems perspectives (e.g., Kahneman, 2011) suggest that risk-taking behaviors like committing a crime involve the operation of two distinct but interconnected systems—one of which is the immediate reward system and is focused on the “here and now”—and a second system that involves rational, deliberate, future-oriented and directed at longer term objectives (Kahneman, 2011). Terms of criminal decision-making, these processes are often described as “hot” and “cool” modes (Van Gelder et al., 2013).

Interestingly, both sexual robbery and sexual burglary had a subgroup of “novices” who presented a more impulsive and opportunistic crime-commission process, indicative of a person whose decision to offend operates primarily on “short-term” rewards or “hot” modes. Thus, it appears that decision-making processes involved in “novice” subgroups were more affected by the “here and now,” as evidenced by opportunism and lack of precautions and detection avoidance strategies. Similarly, we observed subgroups of intermediate offenders, who appeared to be engaging in “myopic” decision-making processes (e.g., Pedneault et al., 2017). More specifically, the crime-commission process of intermediate offenders in both domains was characterized by only taking some precautions to avoid detection in crime phase (e.g., protecting their identity or acting on the victim) and not engaging in more long-term reward strategies (e.g., destroying and removing evidence). Similar to other studies of rationality in sexual crimes (e.g., Pedneault et al., 2017) this suggests that offenders were moderately concerned with detection avoidance, but likely prioritized the need for immediate material gain (i.e., monetary or sexual) over the risks of being detected.

Conversely, “expert” subgroups appear to be oriented toward longer term objectives that are more in line with “cool” modes of decision-making (Kahneman, 2011; Van Gelder et al., 2013). For example, the pre-crime and crime phases of the “expert” sexual robbery subgroup were characterized by more rational and deliberate decision-making (i.e., “target appraisal” skills). This type of decision-making is characterized as more automatic and instantaneous (e.g., Kahneman, 2011) and thus may enable them to “function well” even in domains where they may not have extensive experience (i.e., committing a sexual assault against a stranger). Thus, although sexual robbery “experts” did not show strong evidence of “domain-specific” expertise in sexual offending, some clearly have developed specific violent offense-related skills oriented around their desire to maximize rewards (i.e., monetary, sexual) while minimizing risks of detection.

Finally, the “experts” in sexual burglary represent a unique subgroup who most closely resembles a domain-specific “expert” (i.e., someone who has developed specialized skills in sexual burglary specifically). These are similar decision-making processes that have been described in burglary (Nee & Meenaghan, 2006) and persistent child sexual offending (Bourke et al., 2012; Ward, 1999) and can be reflected in actions taken during the crime-commission process such as planning, identification of targets, conducting risk appraisal, and taking steps to avoid detection throughout the crime-commission process (Nee & Ward, 2015; Ward, 1999). This provides important insight into the underlying decision-making processes involved for “experts” in sexual burglary, which appears to be more future-oriented, deliberate, and directed toward the longer-term objective of avoiding detection for a sexually motivated crime. Nonetheless, all “experts” in the study were eventually caught by the police. It is important to note that this does not necessarily mean that unapprehended offenders would show evidence of greater expertise than our “expert” subgroups, but more so, indicates that offenders are not solely capable of relying on their abilities and skills to avoid detection. Factors such as cognitive biases (Dror, 2011), errors in judgment (Chi, 2006), risky decision-making (Weinborn et al., 2013), and even the offender’s effect prior to the crime (Van Gelder et al., 2013) may all impact the decision-making process during the commission of the crime.

### Practical Implications

In terms of practical implications, our findings highlight the importance of accounting for offense-related competencies to better understand the heterogeneity of offenders. As mentioned by Ward (1999), expertise can facilitate offending behaviors, thus a better understanding of expertise and its cognitive, behavioral, and affective mechanisms are important for understanding the commission of an offense, as well as how future offenses can be prevented. For instance, Bourke et al. (2012) suggest that late-onset or less experienced offenders may be easier to treat because their offense-related knowledge, skills, and interpretation of their offense are not as well established compared with more expert offenders, and thus may be easier to disrupt. This is important to consider in the context of the current findings, as a large proportion of sexual robbery was classified as “novice” (37.8%). Similarly, for sexual burglary, the identification of “novice” subgroup (29.4%) is important because detecting offenders at early stages of their criminal career may be able to encourage desistance before expertise accrues (Nee, 2015). This stresses the importance of breaking down the offense process to micro-decisions and their consequences, as this could aid clinicians in detecting maladaptive coping strategies and areas where poor coping responses may trigger engagement in future offense situations (Bourke et al. 2012).

On the other hand, “experts” in sexual burglary comprise of nearly 20% of our sexual burglary sample. Although stranger sexual burglary offenses are rare, offenders who demonstrate expertise in detection avoidance, like the “experts” in sexual burglary, are also thought to be among the most coercive and controlling subset of interpersonally violent offenders, have better emotional regulation, and the most entrenched and embedded schemes (Fortune et al., 2015). Thus, due to the accumulated expertise, this subgroup may be the most difficult to treat (Ward, 1999). As noted by Ward (1999), “the tendency for some sexual offenders to progress to more violent, intrusive, and severe forms of sexual violence may be partially a function of their increased ability to do so”(pg. 303). Considering the association that sexual burglary has with an escalation in sexual dangerous, to even more violent forms of offending, such as sexual homicide (e.g., Schlesinger & Revitch, 1999; Vaughn et al., 2008) these offenders may represent a significant group for clinical intervention and rehabilitation. Finally, our findings could also help to improve assessment and treatment for sexual crimes that are hybrid in nature. For example, both sexual robbery and sexual burglary had a subgroup that appeared to be primarily sexually motivated and may require different treatment and management needs than opportunistic “novices.” Moreover, sexual robbery experts, and intermediate subgroups characterized by weapon use may be high risk for chronic violent offending. For example, armed burglary has been associated with severe forms of violent offending, such as kidnapping, armed rape, armed, robbery, and first-degree murder (DeLisi et al., 2017). Thus, by examining an offender’s “expertise” practitioners may be in a better position to understand the vulnerabilities or cues that may delay, or prevent, the reoccurrence of offending behavior (Bourke et al., 2012).

## Limitations and Future Research

Although we believe the current study has important implications, some limitations to this study must be noted. The first pertains to the nature of the data. Police data have considerable strengths in that it offers extensive and detailed information pertaining to the victim, offender, and offense. On the other hand, it is limited in its information related to an offender’s developmental and criminal history as well as psychological profiles. In particular, future research may benefit from examining the associations between criminal expertise and psychopathy, particularly among more expert and intermediate-violent subgroups given its association with serious criminal offending (e.g., DeLisi, 2016). As mentioned by Chopin and Aebi (2018), it is also important to note there are some cases where investigators may fail to identify links between cases. As a result, we were unable to determine what role undetected serial offenses may play in expertise. For instance, Park et al. (2008) found that behaviors such as forensic awareness, deterring victim resistance, and completion of a rape were more often found in serial offenders. Of particular interest for future research might be the role that criminal expertise plays in undetected sexual offense, or in sexual murders or serial rapes, which constitute the most serious forms of sexual offending.

Finally, our data focus exclusively on sexual crimes, and as a result, we do not have access to complete criminal histories (unless they have a history of prior sexual violence). Thus, we are only able to determine whether an offender had been charged or convicted for previous offense but do not know details pertaining to whether sexual burglary offenders had a history of robbery or vice versa. As a result, we are unable to determine what stage offenders are in their criminal career, or the full extent that prior offending played in the development of expertise. It is important to note, however, that criminal histories are less relevant for the current study because we are focused on understanding differences in offense-related competencies and not how expertise is developed for each subgroup. Moreover, the inclusion of criminal histories can be problematic, especially for capturing expertise, given the assumption that more expert offenders will be more likely to have undetected offenses (Ó Ciardha, 2015). Similarly, both burglary and robbery offenses have low clearance rates (e.g., Nee, 2015; Smith, 2003) and so the benefits of criminal history data for the study of expertise would likely offer limited utility. Thus, future studies should strive to include data that include both official (i.e., charges and convictions) and unofficial (e.g., self-reported data) sources to build a more complete picture of the role that prior offending plays in criminal expertise.

## Notes

1. We examined solved cases only (*N* =417 unsolved cases excluded) so that we could include offender characteristics and also because we are focused on behavioral manifestations of criminal expertise during the crime-commission process and not the actual outcome of this process (i.e., whether the case was solved or unsolved). We also excluded cases with male victims (*n* = 29) due to differences in the crime commission process observed between male and female victims of sexual crimes (e.g., Beauregard & Proulx, 2007).

2. A contact sexual offense for the purposes of this study includes any vaginal/anal penetration (63.7%), rubbing penis against victim (9.3%), masturbation (19.8%), cunnilingus (6.9%), fellatio (48.5%), foreign object insertion (2.5%), digital penetration (27.2%), fondling (56.2%), and kissing (27.0%). With the exception of cunnilingus, no statistical differences were observed in sexual acts between sexual burglary and sexual robbery offenses.

3. We also tested the date of the offense against each of our subgroups identified through the latent class. None of the latent subgroups of criminal expertise was significantly associated with the date of the offense, with the exception of Group 1 and Group 2 in sexual robbery offenses. In these cases, Group 1 was significantly associated with a later offense date (*M* = 2007.60, *SD* = 2.93) compared with Group 2 (*M* = 2005.39, *SD* = 6.60). All results are available upon request.
